# Annotated dataset of simulated voiding sound for urine flow estimation

**DOI:** 10.1038/s41597-025-05358-1

**Published:** 2025-06-13

**Authors:** Marcos Lazaro Alvarez, Laura Arjona, Alfonso Bahillo, Ganeko Bernardo-Seisdedos

**Affiliations:** 1 Faculty of Engineering, University of Deusto, Bilbao, 48007 Spain; 2 Department of Signal Theory and Communications, Universidad de Valladolid, Valladolid, 47011 Spain; 3 Department of Medicine, Faculty of Health Sciences, University of Deusto, Bilbao, 48007 Spain

**Keywords:** Experimental models of disease, Quality of life, Urinary tract obstruction

## Abstract

Sound-based uroflowmetry is a non-invasive test emerging as an alternative to standard uroflowmetry, estimating voiding characteristics from the sound generated by urine striking water in a toilet bowl. The lack of labeled flow sound datasets limits research for developing supervised AI algorithms. This work presents a dataset of simulated urinary flow sound recordings at flow rates from 1 to 50 ml/s, in increments of 1 ml/s, against water in a real toilet bowl. Flow generation employed an L600-1F precision peristaltic pump, with simultaneous recordings from three devices: high-quality Ultramic384k microphone, Mi A1 smartphone and Oppo smartwatch. Water was expelled through a 6 mm diameter nozzle (simulating the urethra) from a variable height of 73 to 86 cm, mimicking adult urination. The dataset provides 60-seconds labeled, constant-flow audio recordings (WAV format). This resource is intended to support research on sound-based urinary flow estimation by developing and validating supervised artificial intelligence algorithms.

## Background & Summary

The growing development of artificial intelligence (AI) is transforming healthcare systems, steering them towards a more proactive and remote approach, which promises to redefine healthcare at multiple levels. This technological evolution allows healthcare providers to anticipate diseases through predictive analysis based on massive and real-time health data, significantly improving the early detection of pathologies, personalization of treatments and management of chronic diseases^1^. At the same time, for patients, AI systems are facilitating more convenient and continuous access to healthcare, eliminating the barrier of distance and improving treatment adherence through remote monitoring and automated reminders^2^.

One medical test significantly benefiting from AI is sound-based uroflowmetry (SU). This innovative technique seeks to estimate urinary flow patterns during bladder emptying based on the sound generated by urine striking the water surface in a toilet bowl. SU emerges as a remote and proactive alternative to uroflowmetry (UF), a standard clinical test performed by urologists to detect issues associated with urinary tract symptoms (LUTS), such as obstructions or voiding dysfunctions.

UF, while effective, is conducted in a clinical setting where the patient must urinate into a uroflowmeter, a device that measures critical parameters such as urinary flow rate, voided volume and the times involved in the voiding process^3^. However, the effectiveness of UF can be affected by contextual factors, such as the stress or discomfort patients may experience when undergoing the test in an unfamiliar or unnatural environment, potentially altering their normal voiding patterns^4^. Moreover, just one uroflowmetry test could not be representative enough of how the patient usually voids. Therefore, SU improves patient adherence allowing home based interventions and reduces the variability of the results increasing the number of tests.

The main challenge in developing AI-based research related to SU relies on the absence of public datasets with labeled flows. This issue poses a significant challenge for researchers and limits the advancement of associated clinical applications. Currently, there are multiple works in the literature that address the estimation of flow parameters in SU^5–9^, but most researchers create their own databases independently, leading to significant variations in terms of experimental design, recording devices used, flow parameters and characteristics of the subjects under study. This lack of standardization makes it difficult to compare studies, impedes the replicability of experiments and creates inconsistencies in AI based algorithms applied to SU. Moreover, these datasets are not public.

Among the recording devices used in different studies, there is a wide variety, including smartphones^8–10^, dedicated microphones^11,12^ and smartwatches^13,14^, which produce acoustic data with very different characteristics, further widening the gap between technological developments and clinical applications. The heterogeneity in the recording and data processing protocols limits the ability to develop robust and generalizable algorithms that can be validated under various clinical conditions.

The availability of labelled datasets is essential for training AI models capable of accurately predicting urinary flows and detecting pathological conditions earlier. In addition, the SU combined to these AI-based models can be used to create a digital voiding diary that measures multiple flows at different times of the day and night, which could be significantly more useful and objective for determining any pathology. Without well-curated public datasets, the possibility of creating models that can be applied to different devices and clinical scenarios is compromised, limiting the potential impact of SU as an accessible and reliable diagnostic tool. Creating a standardized, public, multi-device dataset, even if based on synthetic flows, could be a key step toward democratizing this technology and its effective integration into clinical practice.

In this work, we have undertaken the task of creating a synthetic flow dataset ranging from 1 to 50 ml/s in increments of 1 ml/s against water in a real toilet, using a L600-1F precision peristaltic pump and recording with three devices: high-quality Ultramic384k microphone, Mi A1 smartphone and Oppo smartwatch. The water was expelled through a 6-mm diameter nozzle that simulates the average external urethral meatus of the adult male^15^, from a variable height of 73-86 cm. This clean dataset, without any other sound but the simulated urine striking the water of a real toilet bowl, could be used as the basement over which voids are simulated in real environments adding background noise before training AI-based models.In the following sections, we will provide a detailed analysis of the steps and methods used to produce the data.

## Methods

### Flow generating device

**Fig. 1:**
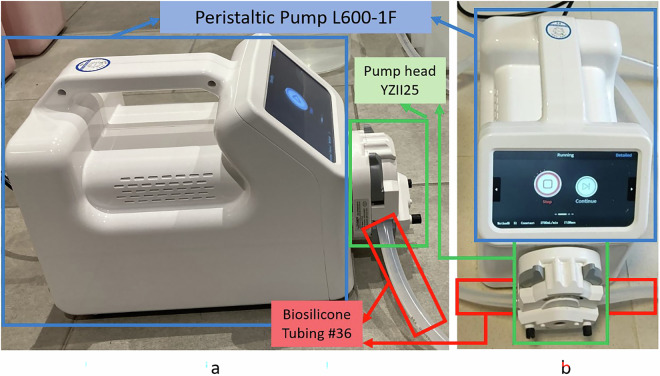
Precise laboratory peristaltic pump (L600-1F) 0,16 *μ*l/min-3000 ml/min: (**a**) side view and (**b**) front view.

For the generation of the dataset, we used a L600-1F precision peristaltic pump^16^, which generates flows in the range of 0.16 *μ*l/min – 3000 ml/min (2.67 nl/s – 50 ml/s), depending on the head and tube diameter used. For our application, we aimed to generate flows between 1 and 50 ml/s, as this is the flow range observed in uroflowmetry tests according to the International Continence Society (ICS)^17^, corresponding to flows of 60 ml/min – 3000 ml/min. In our case, we used the YZII25 pump head with 9.5 mm inner diameter x 2.4 mm wall thickness biosilicone tubing (code *#*36), which covers a flow range of 160 *μ*l/min – 3000 ml/min (see Fig. 1). The length of the biosilicone tubing was 15 m and we used this length to place the peristaltic pump in another room outside the bathroom where the microphones were recording, to isolate the environment from the noise produced by the pump. We had to calibrate the pump using a graduated cylinder to guarantee the flow values supplied by the peristaltic pump, as it will be shown in Section 3, validating the accuracy of the flows.

Table 1 shows the specifications provided by the manufacturer for the L600-1F pump model.

**Table 1:** Specifications for L600-1F Pump Model.

Parameter	L600-1F
**Speed**	0.1 rpm – 600 rpm CW/CCW
**Speed resolution**	0.1 rpm (speed < 100 rpm), 1 rpm (speed > 100 rpm)
**Flow rate**	0.16 *μ*l/min–3000 ml/min
**Dispensing volume**	100 *μ*l–9999 l (±2% accuracy with calibration)

### Audio recording devices

Building on the diversity of recording devices reported in previous SU studies, we selected three representative types to reflect common practices and support methodological consistency. Smartphones are widely used due to their availability, integrated microphones and ease of use^6,8,9^. Dedicated microphones are used in studies that require high-fidelity or privacy-focused acoustic data^11,12^. Smartwatches, on the other hand, enable hands-free, body-fixed recording and are increasingly adopted for long-term, home-based monitoring^13,14^. In particular, the selection of the smartwatch model used in our dataset is supported by the comparative evaluation presented in^13^, which assessed multiple smartwatch devices and justified the use of the Oppo smartwatch for its recording performance and suitability for non-intrusive SU testing.

**Fig. 2:**
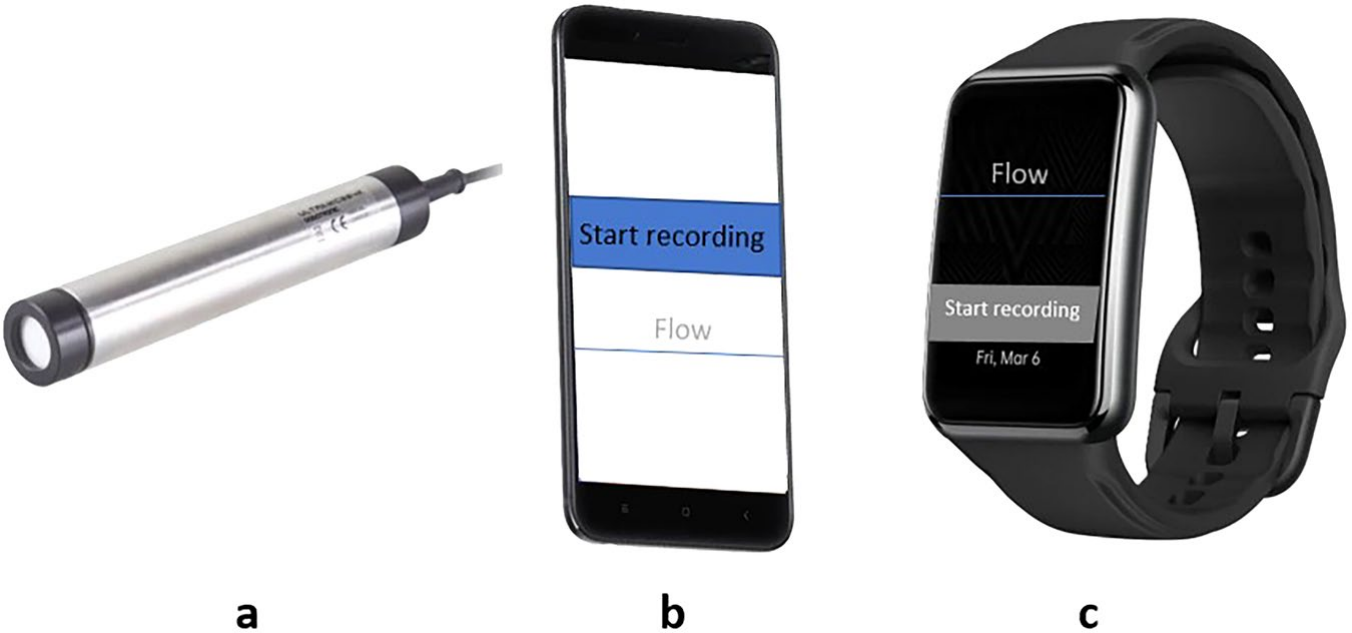
Audio recording devices used: (**a**) UM, (**b**) Phone and (**c**) Watch.

Accordingly, our dataset includes one representative device from each of these categories (smartphone, smartwatch and professional microphone). This selection is intended to capture a broad range of realistic acoustic scenarios while supporting reproducibility and cross-device algorithm development. The selected devices are shown in Fig. 2 and described below:

- Ultramic384K (UM): a high-quality external USB microphone (Ultramic384k by Dodotronic)^18^, incorporating a Knowles FG23629 MEMS sensor. It is capable of a maximum maximum sampling rate (SR) of 384 kHz, allowing for detailed analysis of a wide frequency spectrum. In our tests, we used a SR of 192 kHz, sufficient to analyze up to 96 kHz, while minimizing data volume. The microphone was connected to a laptop where recordings were triggered using a custom Python script with pre-set parameters.
- Mi A1 smartphone (Phone)Phone: a Xiaomi Mi A1 smartphone equipped with an integrated Knowles SPU0410LR5H-QB MEMS microphone, commonly found in mobile consumer devices. The microphone operated at a SR of 48 kHz, capturing frequencies up to 24 kHz. Smartphones have been extensively used in previous SU studies due to their accessibility, built-in microphones and ease of software integration. In our setup, recordings were managed using a custom Android app with fixed parameters.
- Oppo smartwatch (Watch): an Oppo smartwatch integrating a medium-quality embedded microphone (exact chipset not publicly disclosed), validated in prior SU studies^13^. The watch recorded audio at a SR of 44.1 kHz and was selected for its practicality during voiding, offering a fixed recording position on the wrist without interfering with the act. Recording was initiated using a companion Android mobile app.

### Experimental Setup

**Fig. 3:**
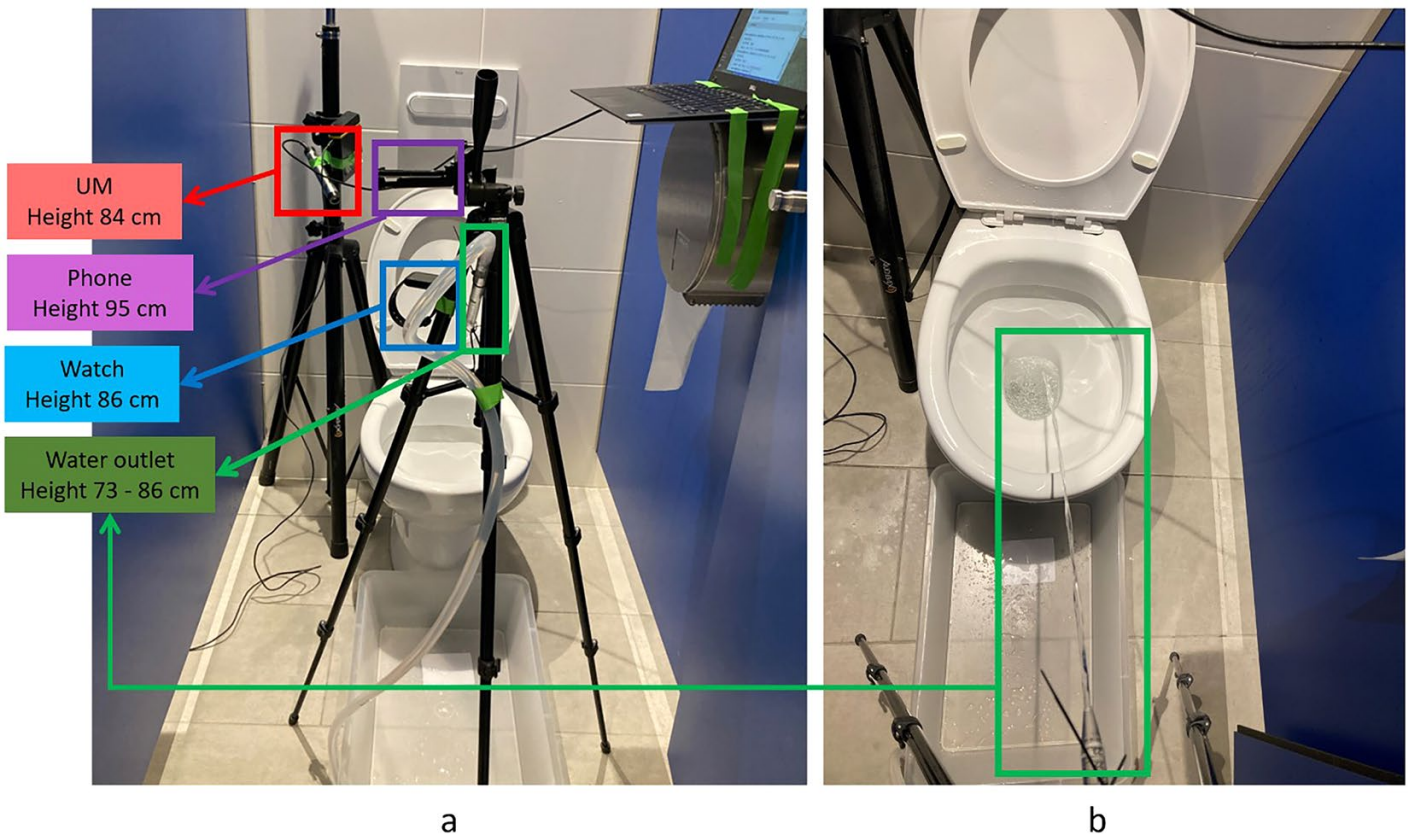
Recording stage: (**a**) front view and (**b**) top view.

Figure 3 shows the position of the recording devices in the bathroom where the flow recording tests were conducted. The heights from the ground for the UM, Phone and Watch were 84, 95 and 86 cm, respectively. The selection criteria for the height and position of the UM and Phone were based on the average position and height of a toilet tank. For the Watch, the position and height simulated the average wrist height from the ground for a person standing with relaxed arms by their sides.

The height of the water outlet nozzle was set at a reference height of 85 cm, which approximates the typical height of the urine stream exit in standing adult males. While the average male hip height is around 90 cm^19^, the anatomical location of the urethral meatus is slightly lower, justifying the use of 85 cm as a realistic reference point for simulating voiding conditions. To ensure that the stream consistently impacted the water surface in the toilet bowl across all flow conditions, the nozzle height was adjusted between 73 and 86 cm depending on flow intensity. At lower flows, a more horizontal jet trajectory was required and the inclination of the outlet tube was also modified accordingly. These variations remained within realistic anatomical limits and were not expected to significantly affect the acoustic properties of the recorded sound.

### Data Acquisition

**Fig. 4:**
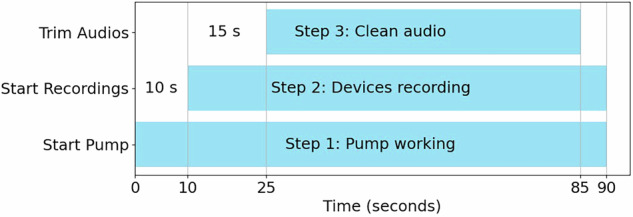
Process Flow Diagram.

**Fig. 5:**
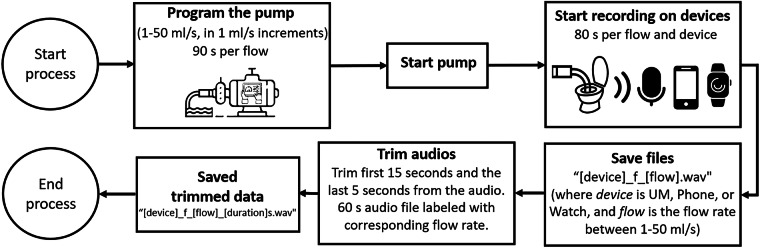
Flowchart describing the audio collection procedure.

Our goal is to obtain labeled audio samples of constant flows with a duration of 60 seconds for each recording device. The data were collected under controlled conditions to minimize background noise and external interference. The pump was programmed for each of the flows from 1 – 50 ml/s in 1 ml/s increments, with a duration of 90 seconds. Once the pump was activated (Fig. 4, Step 1: Pump working), the recording process for each microphone was started activating the UM, Phone and Watch. The recording start process was as follows: for the UM, the “Enter” key was pressed on the laptop keyboard; for the Phone and Watch, a touch button was pressed in their respective Android based mobile apps. All devices were configured to record for 80 seconds once initiated (Fig. 4, Step 2: Devices recordings). The file name format for the recordings of the different flow audios for each device is as follows: “[device]_f_[flow].wav” (where *device* is UM, Phone, or Watch and *flow* is the flow between 1 and 50 ml/s). Once all the audio files were obtained with their respective flow labels for each device, they were trimmed to 60 seconds of pure urination sound with their corresponding flow. For this, we trimmed the first 15 seconds of each audio to remove the initial noise related to the start of recording and the last 5 seconds, finally obtaining labeled 60-seconds audio files (Fig. 4, Step 3: Clean audio). Figure 4 shows a diagram of the progress followed from step 1 to step 3 to obtain the audio recordings from the dataset. The trimmed audio “[device]_f_[flow]_[duration]s.wav,” where the number of seconds in the audio is added under the tag *duration*. Figure 5 shows the flowchart for the audio collection procedure.

We have also included 30 seconds of total silence recording for the three devices in the same environment where all the flow data were recorded, labeled with a flow value of 0.

## Data Records

The audio dataset described in this work has been deposited in the Figshare repository^20^ (10.6084/m9.figshare.27606642) and is organized as follows:

- Three folders named Ultramic_1min, Phone_1min and Oppo_1min, corresponding to the recording devices UM, Phone and Watch, respectively.
- Within each folder, there are 51 audio files in WAV format for each flow rate (1-50 ml/s, in increments of 1 ml/s), as well as an additional file representing 30 seconds of silence.
- Each file includes metadata specifying the flow rate, recording device, SR and duration in seconds, serving as annotations for supervised learning tasks. No additional annotations or manual classifications were applied beyond these flow rate labels.

## Technical Validation

**Fig. 6:**
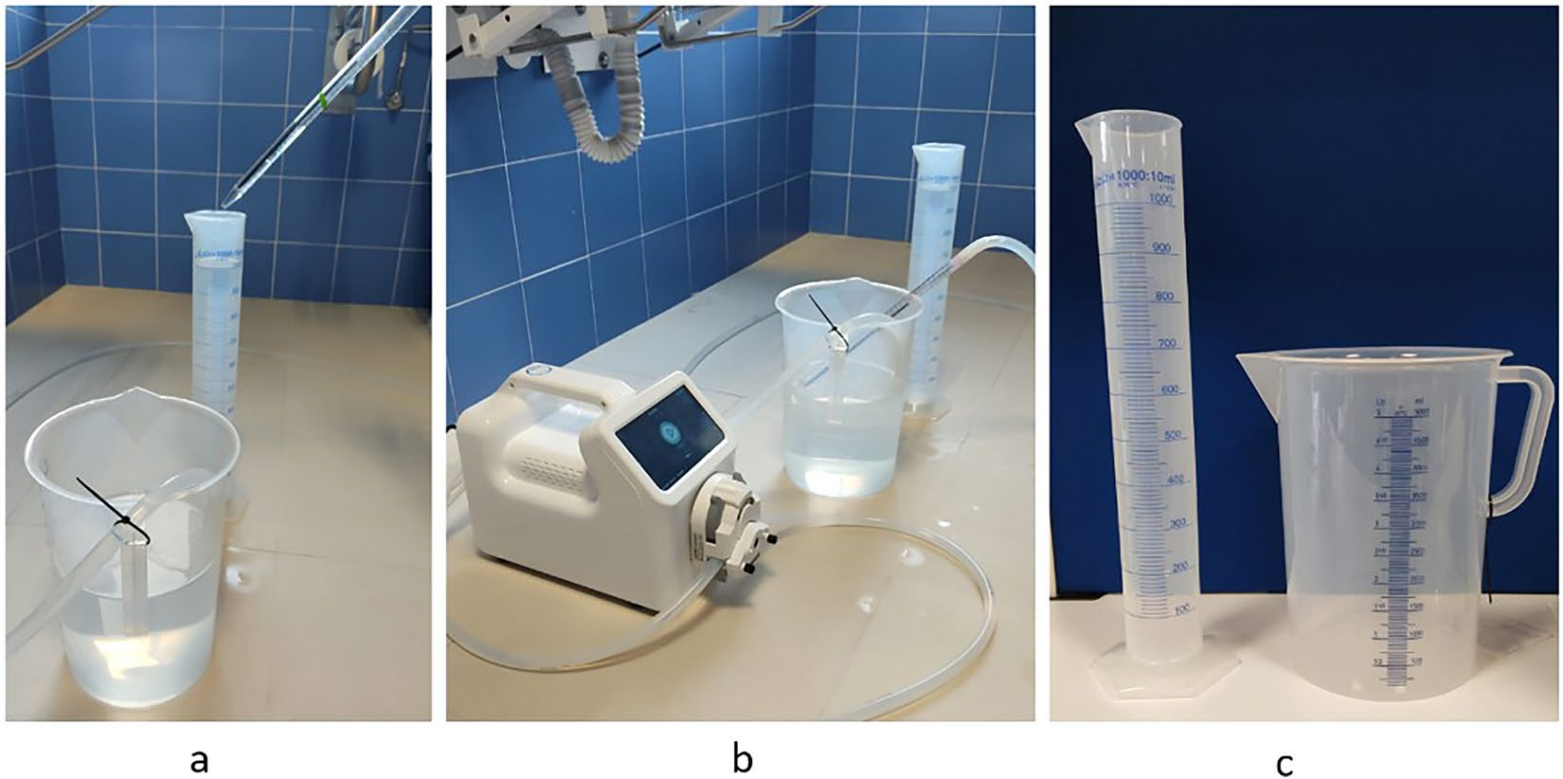
Flow validation scenario with the peristaltic pump: (**a,b**) calibration test setup and (**c**) graduated cylinder.

To validate the quality of the dataset, we conducted multiple trials to ensure consistency in the recordings. The validation of the flow rate supplied by the peristaltic pump was performed using a graduated cylinder of 1000 ml volume with a resolution ±10 ml (see Fig. 6). The results obtained are presented in Table 2. It contains the programmed flow rates, the programmed operation time, the expected volume in ml and the real volume emptied by the pump, as it comes from the factory.

**Table 2:** Validation of the peristaltic pump with times in seconds, expected volumes and actual emptied volumes.

Programmed		Expected	Real volume		Relative error (%)	
Flow	Time	volume	Uncalibrated	Calibrated	Uncalibrated	Calibrated
(ml/s)	(s)	(ml)	(ml)	(ml)		
50	20	1000	1000	—	0.00	—
45	22	990	1000	—	1.01	—
40	25	1000	990	—	1.00	—
35	28	980	960	—	2.04	—
30	33	990	970	—	2.02	—
25	40	1000	990	—	1.00	—
20	50	1000	950	990	5.00	1.00
15	60	900	830	900	7.78	0.00
10	60	600	560	590	6.67	1.67
5	60	300	270	295	10.00	1.00

The validation results confirm that the peristaltic pump has a high degree of accuracy in most of the programmed flow rates, with minimal differences between the expected and actual emptied volumes. For higher flow rates, such as 50 ml/s to 25 ml/s, the relative error percentages are quite low, remaining below 2.1%. However, for lower flow rates, specifically between 20 ml/s and 5 ml/s, the relative errors increase significantly, reaching 10%. These deviations can be attributed to the use of a 15-meter hose, as small pressure losses and internal friction have a greater impact on reduced flow rates. At higher flows, the pump can better compensate for these losses, maintaining a more stable flow.

To reduce errors associated with low flow rates, we calibrated the pump for low flows from 5 to 20 ml/s in increments of 5 ml/s. Table 2 shows the results for the real calibrated volume and their corresponding calibrated absolute errors from 5 to 20 ml/s. In conclusion, the results are consistent and suitable for the purpose of this study, providing a solid foundation for creating a dataset with accurately labelled flows.

The use of the UM microphone provided high-resolution sound data, while recordings from the Phone and Watch represent more accessible alternatives that may be more commonly available for practical applications.

## Usage Notes

Researchers can use this dataset to develop and evaluate AI-based models for estimating urinary flow rates based on sound recordings. Possible applications include training regression models to predict continuous flow rates from acoustic features (e.g., Melfrequency cepstral coefficients (MFCC)), or classification models to categorize flow profiles into clinically relevant groups (e.g., low, normal, high). The labeled structure of the recordings supports supervised learning, model validation and benchmarking in a reproducible manner.

To support reproducibility and practical application of the dataset, a sample Python notebook compatible with Google Colab has been provided as supplementary material in the dataset repository (sound_dataset_processing.ipynb)^20^. The script implements the recommended preprocessing steps, including background noise addition to simulate real environments, noise reduction via high-pass filtering, audio segmentation, MFCC-based feature extraction, normalization across devices to address microphone variability and supervised modeling with 10-fold cross-validation. Additionally, the high sampling rate of the UM allows for a detailed analysis in the frequency domain, which could provide deeper insights into the acoustic properties associated with different flow rates.

As a limitation, it should be noted that the dataset was created under controlled conditions simulating a standing adult male with relaxed arms, at a fixed height of approximately 85 cm. Variations in toilet structure or anatomical factors (including gender differences) and voiding posture (sitting or standing) may influence the acoustic characteristics of real voiding events. Researchers should take these factors into account when generalizing findings to clinical settings.

## Data Availability

The Python notebook sound_dataset_processing.ipynb has been developed to support the preprocessing and modeling of the dataset. The notebook is available in the dataset repository^20^ and demonstrates preprocessing, feature extraction, normalization across devices and supervised modeling in a reproducible manner.
